# Study of the Degradation of a TPS/PCL/Fique Biocomposite Material in Soil, Compost, and Water

**DOI:** 10.3390/polym15193952

**Published:** 2023-09-30

**Authors:** Fabián Steven Mosquera Rodríguez, Alejandro Quintero Vélez, Estivinson Córdoba Urrutia, Howard Ramírez-Malule, Jose Herminsul Mina Hernandez

**Affiliations:** 1 School of Chemical Engineering, Universidad del Valle, Calle 13 No. 100-00, Cali 760001, Colombia; mosquera.fabian@correounivalle.edu.co (F.S.M.R.); alejandro.quintero.velez@correounivalle.edu.co (A.Q.V.); 2 Group Investigación en Ciencia Animal y Recursos Agroforestales, Universidad Tecnológica del Chocó, Carrera 22 No. 18B-10, Quibdó 270001, Colombia; estivinson.cordoba@utch.edu.co; 3 School of Materials Engineering, Group Materiales Compuestos, Universidad del Valle, Calle 13 No. 100-00, Cali 760001, Colombia

**Keywords:** degradation, thermoplastic starch, polycaprolactone, biocomposite, fique fibers, microorganisms

## Abstract

The degradability of the biocomposite produced from a binary mixture of thermoplastic banana starch (TPS) and polycaprolactone (PCL) reinforced with fique fibers (Fs) was evaluated in three different environments (soil, compost, water). An experimental design with two factors (soil and compost) and three levels (5, 10, and 20 cm) was used, with additional tests for a third aqueous environment (water from the lake of the Universidad del Valle) at a depth of 20 cm. The biocomposite was prepared from the implementation of a twin-screw extrusion process of the binary mixture TPS/PCL and fique fibers (54, 36, and 10% composition, respectively), followed by hot compression molding, and after that, generating ASTM D638 type V specimens using a stainless-steel die. The specimens were dried and buried according to the experimental design, for a total experimental time of 90 days, and removing samples every 30 days. After 90 days, all samples showed signs of degradation, where the best results were obtained in the compost at a depth of 20 cm (34 ± 4% mass loss and a decrease in tensile strength of 77.3%, which indicates that the material lost mechanical properties). TPS was the fastest disappearing component and promoted the degradation of the composite material as it disappeared. Finally, the aqueous media presented the lowest degradation results, losing only 20% of its initial mass after 90 days of the experiment, being the least effective environment in which the biocomposite can end up.

## 1. Introduction

The high accumulation of solid waste is one of the main problems for the environment today [1,2]. In Colombia, about 11.6 million tons of solid waste are generated annually, and a large part of this waste is single-use plastic, including bags, packaging, straws, and bottles made of fossil material. According to the Colombian Ministry of Environment and Sustainable Development, a large percentage of this plastic waste ends up in landfills and water sources [3], contributing to pollution due to the time it takes to degrade.

On a global scale, the situation is not improving; according to the Organization for Economic Co-operation and Development (OECD), global plastic production doubled from 2000 to 2019, adding 353 million tons (Mt) to the existing ones. Approximately two-thirds of this waste comes from plastic with a life cycle of less than five years, 40% of which comes from packaging, 12% from consumer goods, and 11% from clothing and textiles. At the end of their useful life, only 9% of plastics are recycled, 19% are incinerated, 50% are used in landfill, and 22% are not properly managed, being incinerated in open pits or ending up in land or water-based landfills. In 2019, 6.1 Mt of plastic waste leaked into aquatic media, and 1.7 Mt reached the oceans [4].

In accordance with the above, and to develop planet-friendly alternatives, numerous studies have been carried out on biocomposite and/or bioplastics, which seek to take advantage of biological material to replace conventional plastics [5,6,7,8,9,10,11,12,13,14,15,16,17,18], generating products with similar characteristics (high strength, durability, and robustness, among others), but with shorter degradation times and cleaner processes. However, the bioplastics industry faces challenges, such as high production costs, limited availability of raw materials, inferior mechanical properties compared to conventional plastics [19,20], the need for high-temperature composting facilities, and competition for land use. However, despite these challenges, the industry is growing and can contribute to global sustainability; as proof of the above, the production of bioplastics increased by 30% from 2015 to 2022 (being in 2015 1.7 Mt and reaching 2.217 Mt worldwide by 2022) [21,22].

Polycaprolactone (PCL) and starch blend fall within the group of biodegradable bioplastics; according to the latest data compiled by European Bioplastic in cooperation with the Nova-Institute, in 2020, over 20% of global production (443.1 thousand tons) corresponded to them, and these percentages are projected to grow by 8% by 2025, based on global production of 2.87 Mt of biopolymers. In addition, these biocomposites present a wide range of physical properties that make them attractive in the development and improvement of products, which, combined with other materials, can increase their physical characteristics and applications.

Due to the above, different works have been devoted to the study and development of substitutes for conventional plastics [23,24,25,26,27]. Mina et al. [28] have developed biocomposites based on a thermoplastic starch/polycaprolactone (TPS/PCL) matrix reinforced with fique fibers, focusing their work mainly on physicochemical, mechanical, and thermal characterizations, indicating the need to carry out degradability studies on these materials. In this sense, not necessarily all bio-based materials can be decomposed in microbiological processes; some may require specific conditions for their degradation, and, in case these conditions are not met, the permanence time of this biocomposite would increase, and it would not represent an efficient alternative to existing fossil materials.

Based on the above, the objective of this work is to evaluate the degradation of a biocomposite based on a thermoplastic banana starch/polycaprolactone (TPS/PCL) matrix reinforced with fique (F) fibers, in different media (soil, compost, and water), to obtain more information about the decomposition times under different environmental conditions. Thus, specific processes could eventually be developed to mitigate the environmental impact generated by the use and application of this material in consumer goods or others.

## 2. Materials

The native starch used to elaborate the TPS was obtained from the dried banana root of the Dominico Harton variety [29], from the Asociación de Productores de Finca Tradicional del Norte del Cauca (ASPROFINCA), located in the municipality of Villa Rica (Cauca, Colombia). The glycerol used as a plasticizer, from Químicos del Valle Uno A S.A.S. in Cali, Colombia, is of industrial grade, with a purity of 99.8%. The fique fiber was obtained from a market in the same city and was produced by the company Empaques del Cauca in the city of Popayan, Colombia; the material was used as reinforcement and was cut to an average length of 5 mm. The PCL used was acquired from Perstorp UK Limited of Warrington, UK, under reference CapaTM 6800, with a melting temperature (Tm) of 58–60 °C, moisture content < 1%, and an elongation at break of 800%. The commercial compost was purchased from Vivero Pasoancho in Cali, Colombia, and the soil and water used were extracted from an area near Villa Solar and the Lake Biology experimental station, respectively, located at the Universidad del Valle, Meléndez campus.

## 3. Experimental Procedure

### 3.1. Statistical Experimental Design

A 3^2^ factorial design was used to evaluate the degradation of the biocomposite (TPS/PCL/F) in soil and compost during a period of 3 months; additionally, for comparison purposes, the tests were carried out in an aqueous media, for a single level. The response variable selected for the statistical analysis was the percent mass loss (%ML) of the biocomposite. The analysis of variance (ANOVA) with α = 0.05 was performed using Minitab 18^®^ software (Minitab Inc., State College, PA, USA), and to see the effects and interactions of the factors, the following were used: main effects diagram, contour plot, and surface plot. The factors and levels studied are shown in Table 1. This methodology was developed based on a bibliographic review of Rodríguez, et al. [30], Minchola [31], and Accinelli, C. et al. [32].

### 3.2. Selection, Sampling, and Characterization of Degradation Media (Soil, Compost, or Water)

**Figure 1:**
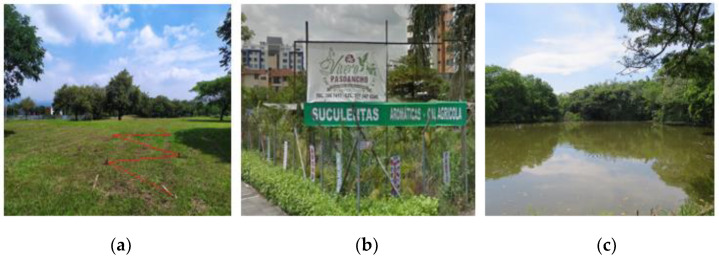
Areas selected for sampling and collection of degradation media: (**a**) soil; (**b**) compost; and (**c**) water.

To simulate the degradation of the biocomposite in the possible environments to which it would be exposed at the end of its useful life, soil and water samples were taken at the Meléndez campus of the Universidad del Valle, while the compost was purchased at the Pasoancho greenhouse (Figure 1). On one hand, each of the media samples was characterized by physicochemical analyses, such as moisture, soil texture, total organic matter, total nitrogen, pH, electrical conductivity, salt content, and microbial activity, in the case of compost and soil. On the other hand, the following were taken for the water sample: pH, electrical conductivity, microbial activity, chemical oxygen demand (COD), oxide reduction potential (ORP), and dissolved oxygen (DO). The analyses were carried out at the LASA agricultural water and soil laboratory of the School of Sanitary and Environmental Engineering, in accordance with the soil laboratory analytical methods proposed by the Agustin Codazzi Geographic Institute [33], and the methods of physical and chemical analysis of water quality [34].

### 3.3. Production of Thermoplastic Banana Starch

The banana starch, previously dried at 60 °C for 24 h, was pre-mixed with glycerol at a ratio of 65 starch/35 glycerol (by mass), using a KitchenAid professional (KitchenAid^®^, Troy, OH, USA) mixer for 10 min, until the material was homogeneous and free of lumps. Then, the material was stored in polypropylene bags for 48 h. The resulting mixture was plasticized in a HAAKE twin-screw extruder machine (HAAKE Polylab OS RheoDrive 7, Thermo Scientific Inc., Waltham, MA, USA), using a temperature profile of 110, 110, 120, 120, 125, 125, 125, 135, 135, 140, 140, and 145 °C for the ten heating zones of the cylinder and of the head. Finally, the obtained TPS was pelletized using a blade pelletizer (Thermo Scientific Inc, Saint Louis, MO, USA).

### 3.4. Preparation of the Binary Mixture TPS/PCL

The pelletized TPS was manually mixed with the PCL at a ratio of 40 TPS/60 PCL (by mass) and subjected to an extrusion process, using the same equipment previously used to obtain the TPS. The temperature profile handled was 122, 124, 124, 126, 128, 130, 130, 130, 132, 132, 134, and 134 °C for the ten heating zones of the cylinder and of the head. Finally, the TPS/PCL binary mixture was pelletized.

### 3.5. Preparation of the Biocomposite

The TPS/PCL binary mixture was combined with the fique fibers at a ratio of 90 TPS-PCL/10 F (in mass), through an extrusion process following the same transformation conditions previously used to obtain the TPS/PCL binary mixture. Plates (composition: 54% PCL, 36% TPS, and 10% fibers) of 1 mm thickness were obtained with the biocomposite material by means of a hot-compression molding process, using 20 ± 0.05 g of material in a mold holder arranged in a semi-automatic hydraulic press Carver MH 4389-4021, with heating and cooling systems by water circulation (Carver Inc., Wabash, IN, USA): the pressure used was 3000 psi at a temperature of 170 ± 5 °C, and a heating-only time of 8 min was used, followed by another 8 min of heating under pressure. With the prepared plates and using a metal die, V-type pro-betas were obtained with the dimensions specified in the ASTM D-638-14 [35].

### 3.6. Degradation–Assembly Configuration

**Figure 2:**
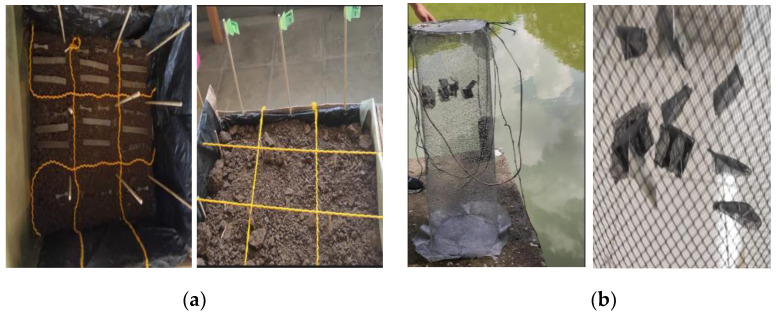
Degradation setup: (**a**) soil and compost and (**b**) water.

Wooden baskets of 53 × 48 × 35 cm in length, width, and height, respectively, were used in the set-up configuration. The degradation media, soil (preserving the depth profile), and compost were placed there. The samples necessary for a period of 90 days were placed equidistantly at the three depth levels (5, 10, and 20 cm), as shown in Figure 2a. In the case of water, the samples were placed inside a sample holder at a depth of 20 cm and protected by a wire-mesh cylinder, as shown in Figure 2b.

### 3.7. Analytical Characterization of Samples

#### 3.7.1. Fourier-Transform Infrared Spectroscopy (FT-IR)

For the determination of the main functional groups in the biocomposite material, PCL, and TPS, a Jasco spectrometer (FT/IR-4100) Type A (JASCO Manufacturing, Portland, OR, USA), operated at 100 scans and a resolution of 4 cm^−1^, was used. Attenuated total reflectance (ATR) methodology was employed using an ATR PRO450-S accessory (JASCO Manufacturing, Portland, OR, USA).

#### 3.7.2. Scanning Electron Microscopy (SEM)

To observe the surface morphology of the TPS/PCL/F biocomposite samples, a JEOL SEM scanning electron microscope model JSM-6490 (JEOL Ltd., Akishima, Tokyo, Japan) operated at 20 kV was used, where the samples were previously coated with a layer of gold using a Denton Vacuum Desk IV (Denton Vacuum, Moorestown, NJ, USA) cold spray coater model STANDAR. A PHENOM electron microscope model PROX (ThermoFisher Scientific, Waltham, MA, USA) operated at 15 kV, without any coating, was also used. Both devices were used to perform measurements at 500 magnifications.

#### 3.7.3. Tensile Test

The tensile mechanical properties of the TPS/PCL/F biocomposite samples were determined for different sampling times (30, 60, and 90 days), at depth levels of 5, 10, and 20 cm. The tests were performed using a Tinius Olsen model H50KS (Tinius Olsen, Philadelphia, PA, USA) universal testing machine, with a 10 KN load cell and wedge-type grips. Type V specimens were used at a jaw displacement rate of 5 mm/min, in accordance with ASTM D-638 [35].

#### 3.7.4. Mass Loss

Samples were previously dried at 40 °C for 24 h in a LabTech LDO-150F (Labtech S.R.L., Sorisole, Bergamo, Italy) forced convection oven, and the mass of the sample was determined before being subjected to the degradation assembly (*M_I_*). Every 30 days, samples were removed from the degradation media, carefully cleaned with distilled water, and then dried at 40 °C for 24 h to record their mass (*M_F_*). Finally, the model presented in Equation (1) was used to calculate the corresponding mass loss (*ML*).

(1) $$\%ML=\left(\frac{M_{I}-M_{F}}{M_{I}}\right)\times 100$$

where:

*M_I_* = Initial mass of dry samples.
*M_F_* = Final mass of dry samples subjected to the degradation environment.

## 4. Results and Discussions

### 4.1. Characterization of Degradation Media (Soil, Compost)

Table 2 shows the results obtained in the characterization of the soil and compost used as degradation environments.

Considering that soil is a system that integrally relates chemical, physical, and biological factors, the results were analyzed to determine the behavior of the media (soil and compost). Based on the pH values of the samples, they were classified according to the information reported by Jaramillo [36] in Table S1: with the above, the soil sample was classified as “strongly acidic”, while the compost sample was classified as “very strongly acidic”. Based on the above classification and according to the intervals established by the USDA in 1971 (Figure S1 [37]), the availability of nutrients for a strongly acid soil was high, compared to a very strongly acid soil, which can be confirmed by the values obtained for the percentage of nitrogen for the soil and compost (1.096 and 0.461%, respectively).

With the results of electrical conductivity, the samples can be classified as “non-saline soil” due to the low content of salts present, as established by the Food and Agriculture Organization of the United Nations (FAO) (Table S2 [37]), so it has a negligible salinity effect.

**Figure 3:**
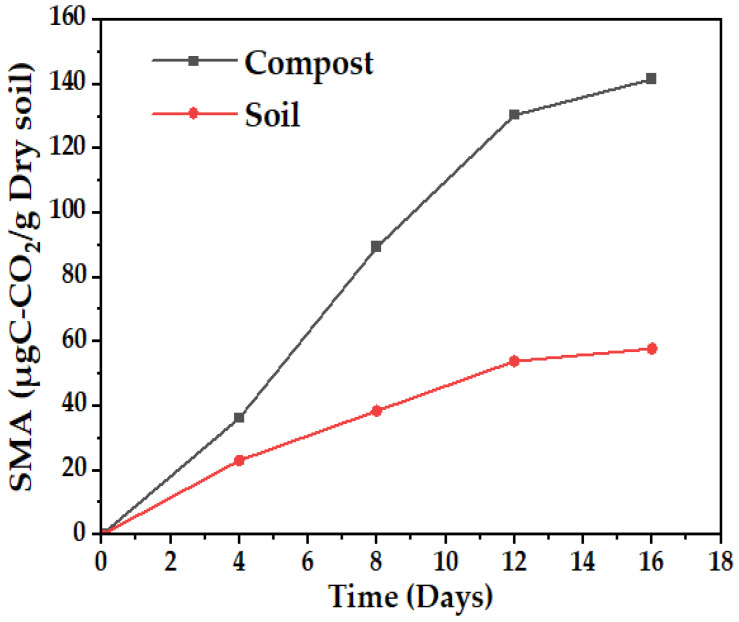
Soil microbial activity (SMA), soil sample, and compost.

Microbial activity in the compost is more than double that of the soil, according to Figure 3, a behavior that can be explained because of the higher amount of organic matter present in the compost (12%) compared to the soil (5.075%), which represents the consumable biomass for the microorganisms present, and although there is a lower amount of nitrogen in the compost, it is more available, according to Figure S1, where the solubility of different nutrients based on pH is related.

Finally, in the case of texture analysis, this allows establishing the capacity of soil to maintain moisture, retain and release ions, and see the availability of nutrients and establishing its aeration capacity and permeability, characteristics that are due to its composition (clay, sand, and silt), considering the proportions receive a classification according to the triangle of textures presented in Figure S2. On one hand, the soil sample is classified as clay loam: its composition contains mostly clay and sand at 38.25 and 38%, respectively, which gives it qualities such as high moisture retention, as well as adhesion and stickiness. On the other hand, the compost sample is classified as sandy loam, with a majority composition of sand (66%): its particles are fine, which makes it saturated with little water, and it does not mold easily, dries quickly in the air, and is not sticky, which allows good aeration. In addition, loam soils are characterized by being fertile, with a high content of organic matter and minerals.

According to all of the above, it can be said that the selected media have good characteristics that would potentially favor the degradation of the biocomposite material.

In the characterization of the aqueous media (Table 3), several conditions were found that allow the proliferation of microorganisms that, together with the movement and friction processes generated by the agitation of the water, can help the decomposition of the biocomposite [38]. The optimum pH range for bacterial growth is between 6 and 8.5, and the sample presents a value of 6.62; therefore, it is an important factor that allows microbial growth, which can be observed in the presence of total coliforms and the number of colony-forming units of mesophilic organisms. Additionally, the values of dissolved oxygen, ORP, and COD are within life-supporting values for a large number of species [39,40].

### 4.2. Analytical Characterization of Samples

#### 4.2.1. FT-IR Spectroscopy

**Figure 4:**
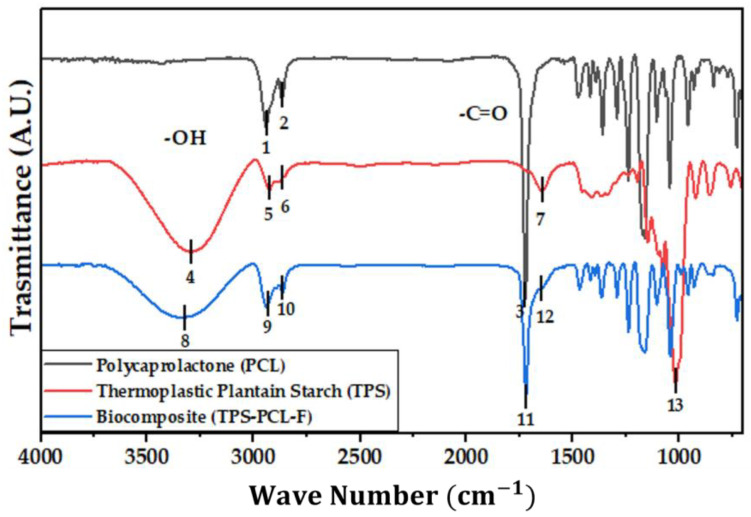
FTIR for PCL, TPS, and TPS/PCL/F biocomposite.

**Figure 5:**
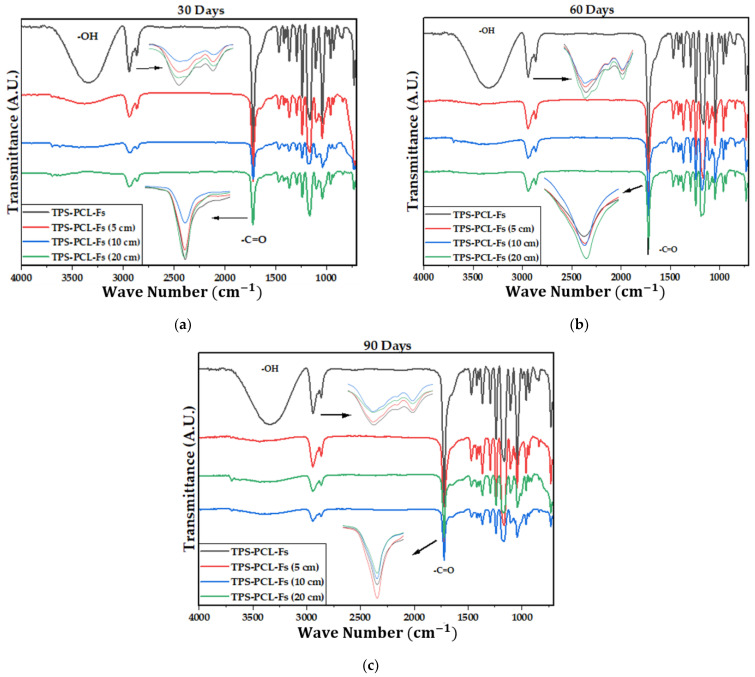
FTIR for TPS/PCL/F biocomposite in soil at (**a**) 30 days, (**b**) 60 days, and (**c**) 90 days.

**Figure 6:**
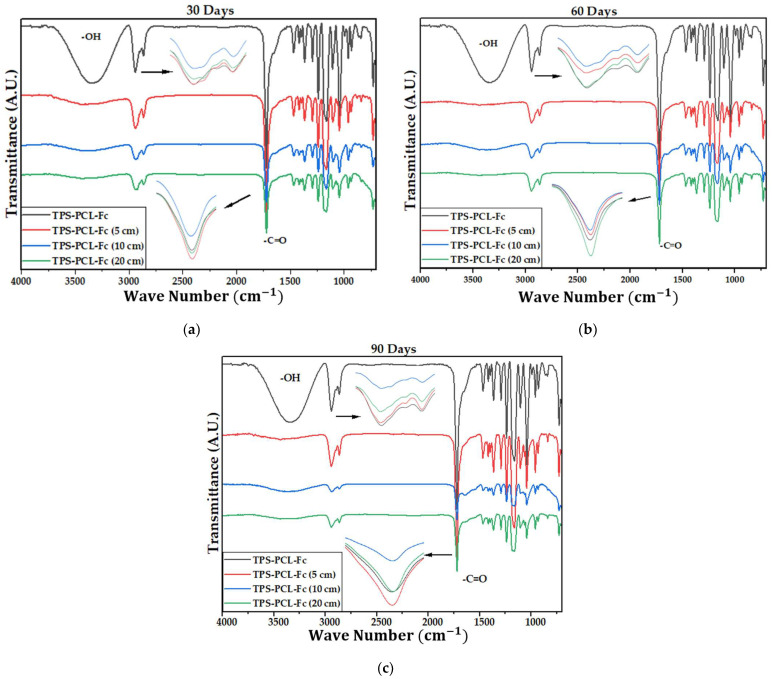
FTIR for the TPS/PCL/F biocomposite in compost at (**a**) 30 days, (**b**) 60 days, and (**c**) 90 days.

**Figure 7:**
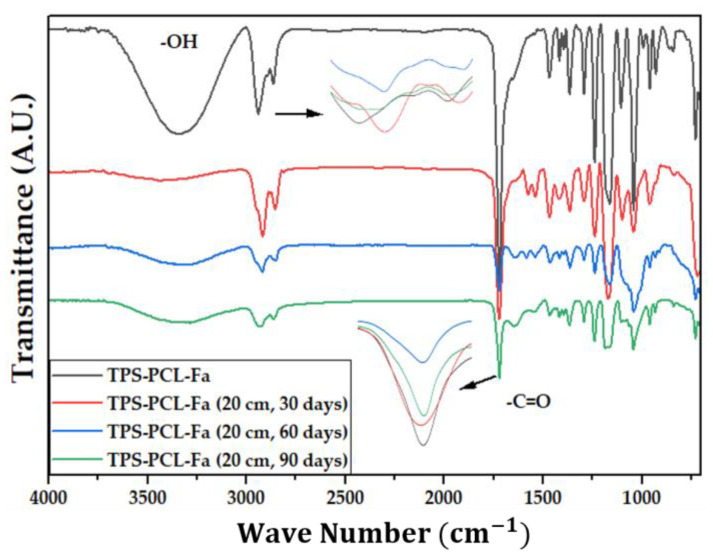
FTIR for TPS/PCL/F biocomposite in water at 20 cm at 30, 60, and 90 days.

Figure 4 shows the IR spectra of the PCL, the TPS, and the biocomposite material (TPS/PCL/F). The last IR spectra are the reference for analysis of this study. In Table 4, the bands of higher intensity are related to the types of vibrations associated with the functional groups present [41,42]. Figure 5, Figure 6 and Figure 7 show the FTIR spectra of the biocomposite samples in the three degradation media at different depths (5, 10, and 20 cm), in an evaluation period of 3 months.

Signal 11 (1721 cm^−1^) in the IR spectrum of Figure 4 corresponds to the stretching in tension of the carbonyl group of the polycaprolactone. This signal had no shift with respect to that found for PCL alone (signal 3), which suggests that hydrogen bonds were probably not significantly formed in the TPS/PCL mixture, thus indicating that it is predominantly immiscible, similar to what was previously reported by [41], who also worked with a binary mixture of TPS/PCL. However, a shift towards a higher wave number of signal 8 was evidenced, related to the stretching of the OH bond, so it can be inferred that intermolecular interactions were probably generated between the hydroxyl groups of TPS and cellulose from F fibers. This type of interactions between TPS and fibers from different sources (sisal, wood, straw, banana leaf, among others) has already been commented on in investigations carried out by Lubis M. et al., Jumaidin et al., and Wang et al. Additionally, Lubis et al., mention that the free hydroxyl groups of TPS that are mutually attracted to the hydroxyls of the macromolecular chain of the fiber are produced during the compression molding process, and, if the displacement is made at a lower wave number, the hydrogen bonds generated are more stable [43,44,45].

It can be observed in the signals belonging to the symmetric and asymmetric stretching of the (-CH_2_) bond at 2941 and 2865 cm^−1^ (Figure 5, Figure 6 and Figure 7) a greater decrease in the intensity of the bands at depths of 10 and 20 cm, compared with that of 5 cm. In addition, the band of the carbonyl group of the polycaprolactone at 1721 cm^−1^ does not undergo considerable modifications; it only presents a small decrease in the band intensity (it had an average decrease in its intensity of 6, 10, and 35% in soil, compost, and water, respectively, compared to the IR spectra of the day 0, black colored). However, there is no displacement in the wave number. This behavior is because PCL degrades slowly due to its high degree of crystallinity and low moisture absorption; therefore, it remains almost unchanged during the 3 months of the test. Nevertheless, the higher the humidity, the greater the decrease in the intensity of the carbonyl bond (CO), probably due to hydrolytic degradation [46].

Additionally, as shown by the infrared spectra in Figure 5, Figure 6 and Figure 7, the representative signal of TPS (band from 3000 to 3600 cm^−1^ due to the stretching of the OH bond) decreased almost completely from the first 30 days of the test (at the end of the 90 days of testing, the intensities of these bands decreased on average by 83% for soil and compost and 66% in water). This is due to the susceptibility of TPS to degradative extraction and to the fact that it presents little stability when humidity conditions are high, added to the fact that the fibers of the biocomposite act as a support for the attack of microorganisms, which begins to generate channels in the matrix of the system that allow the entry of water and favor the disappearance of the plasticized starch more easily [47,48,49].

#### 4.2.2. Morphological Analysis

**Figure 8:**
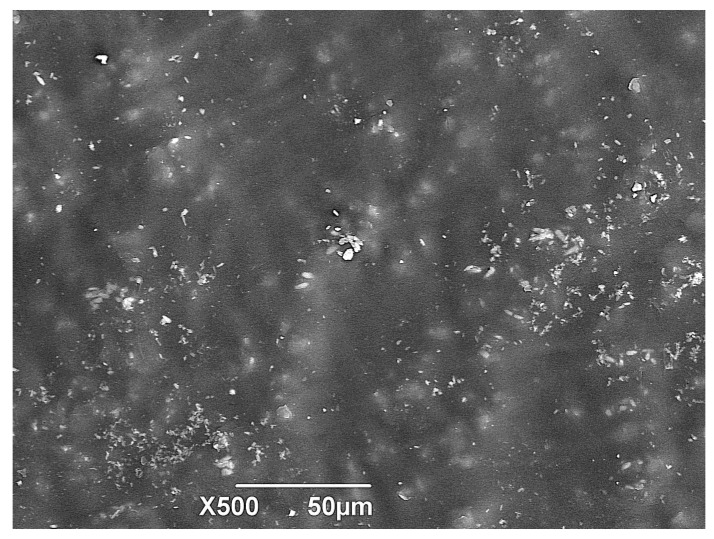
SEM micrograph of TPS/PCL/F Day 0 (standard sample).

**Figure 9:**
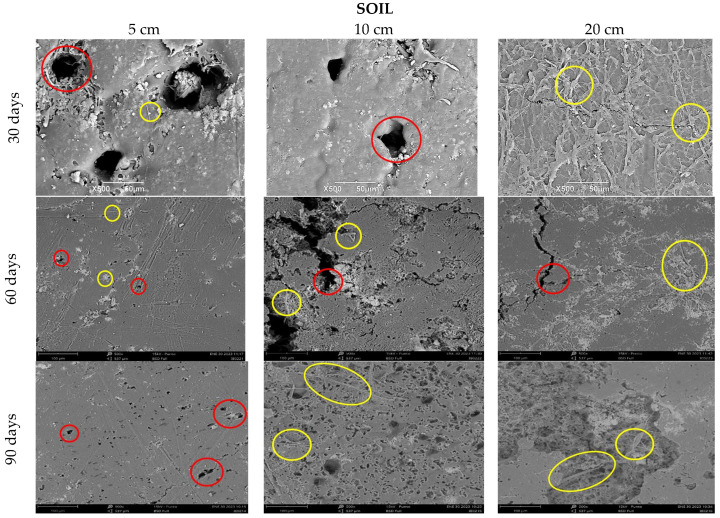
SEM micrograph of TPS/PCL/F in soil at three depth levels (5, 10, and 20 cm) at a time of (30, 60, and 90 days).

As the degradation process of the biocomposite advances, there is a change in the surface of the samples. In Figure 8, it can be observed that the initial samples had a mainly smooth surface, without perforations, being this the reference image to contrast with the results of the materials after conditioning at 30, 60, and 90 days in the different media (soil, compost, and water) and depth levels (Figures 9, 11, and 12).

**Figure 10:**
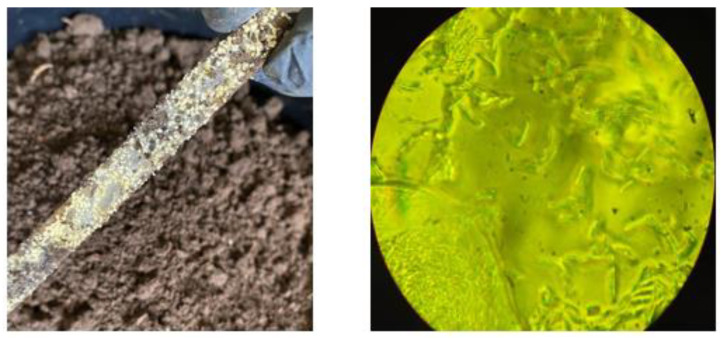
Photographic evidence of the presence of a fungus on the surface of the biocomposite; microscopic image of the fungus at 100× magnification.

Figure 9 clearly shows the deterioration of all the samples buried in the soil, compared to the reference image (Figure 8). For the first 30 days, at depths of 5 and 10 cm, there is evidence of loss of homogeneity in the surface and the presence of quite visible holes (red circles), in contrast to the depth of 20 cm, which can be attributed mainly to microbial action and fungal growth that were perceived when the samples were obtained (Figure 10). The characteristics of the worked clay loam soil allow the formation of conglomerates, and, as a consequence, its humidity conditions can vary at a few centimeters distance, since the soil dries more easily towards the surface and retains more water as the depth increases, generating a distinction in bacterial growth, which could explain the differences in the micrographs at 5, 10, and 20 cm depths, where the fibers of fique at the last level are more exposed (yellow circles, Figure 9). When the analysis is made for 60 and 90 days, it is observed that the external damage increases, with the presence of holes and craters, which means the surface was eroded again due to the microorganisms and the deterioration of the material, given the humidity conditions of the media.

**Figure 11:**
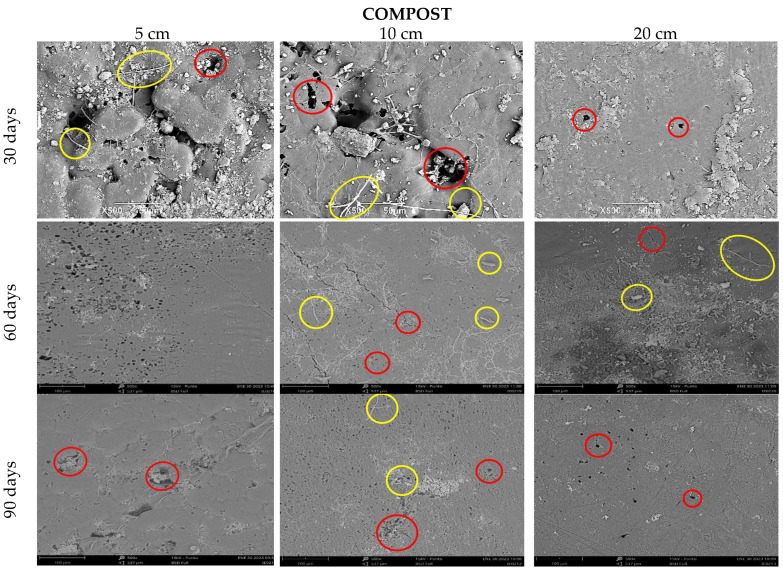
SEM micrograph of TPS/PCL/F in compost at three depth levels (5, 10, and 20 cm) at a time of (30, 60, and 90 days).

Figure 11 shows the deterioration of the samples buried in the compost; as in the soil at the first 30 days, erosion is evident at 5 and 10 cm depths, there are holes (red circles), and the fiber begins to be visible (yellow circles), and at 20 cm, there seems to be less superficial damage. Campos A. et al. characterized a TPS/PCL matrix with 10% sisal fibers and studied its degradation. In their micrographs, they found the formation of agglomerates and the superficial appearance of fibers as a result of the poor interfacial adhesion of the matrix fiber, which contributed to the degradation of their mixture [50,51].

**Figure 12:**
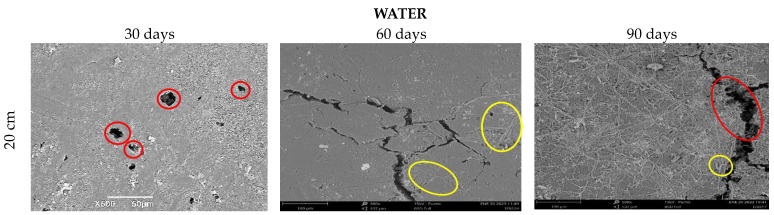
SEM micrograph of TPS/PCL/F in water, 20 cm depth at a time, of (30, 60, and 90 days).

In the case of the aqueous media (Figure 12), the samples were kept at the same depth (20 cm), and thanks to the hydrolytic degradation, it is observed that the fibers are gradually exposed from 30 to 90 days of conditioning. Likewise, holes can be seen on the surface, probably due to the fact that the contact of the polymer with the aqueous media promotes water molecules penetrating into the matrix of the material, causing its swelling, rupture of the hydrogen bonds, and finally the hydrolysis of the unstable bonds, which ends up weakening the biocomposite material [52]. The images obtained with SEM equipment do not provide quantitative information about the degradation process.

#### 4.2.3. Tensile Strength

**Figure 13:**
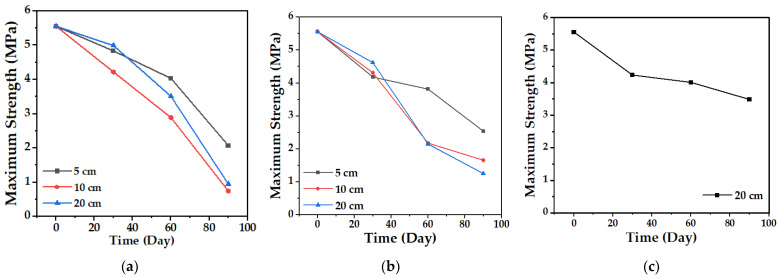
Maximum biocomposite tensile strength (TPS/PCL/F) over a period of 90 days in the degradation media: (**a**) Soil; (**b**) Compost; and (**c**) Water.

**Figure 14:**
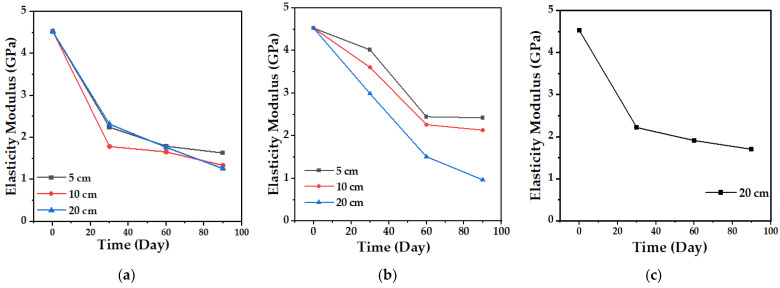
Tensile modulus of elasticity of the biocomposite (TPS/PCL/F) over a period of 90 days in the degradation media: (**a**) Soil; (**b**) Compost; and (**c**) Water.

The tensile test allows establishing the mechanical behavior of the biocomposite material and its change over time. Figure 13 and Figure 14 show the variation in the maximum strength and the tensile modulus of elasticity of the TPS/PCL/F biocomposite, in the three degradation media, during the time of the experiment. The maximum strength presented, as expected, a progressive decrease over time, which was less marked in the case of conditioning in water in comparison with soil and compost at the three depth levels, showing the greatest changes for 10 and 20 cm. In the case of the modulus of elasticity, a decrease occurs, but with minimal changes, having more influence in the cases of soil and compost. When the TPS/PCL binary mixture is made, its processability and biodegradability improves, and the material has hydrophobic characteristics, decreasing plasticization by water absorption [53,54]; but, as described above, as degradation occurs, the structural damage in the surface morphology of the biocomposite increases (Figure 9, Figure 11 and Figure 12). This is possibly due to the loss of mass, allowing the absorption of water inside the TPS due to its hydrophilic nature, which can enable the action of the retrogradation process, allowing the rearrangement of the polymeric chains by the presence of OH groups, followed by recrystallization that increase the stiffness, altering the mechanical properties, decreasing the elongation, and affecting the quality of the biocomposite material, as described in the studies reported by Villada et al. [48], Mina [55], Cortes et al. [56], and López et al. [57].

#### 4.2.4. Mass Loss (%ML)

**Figure 15:**
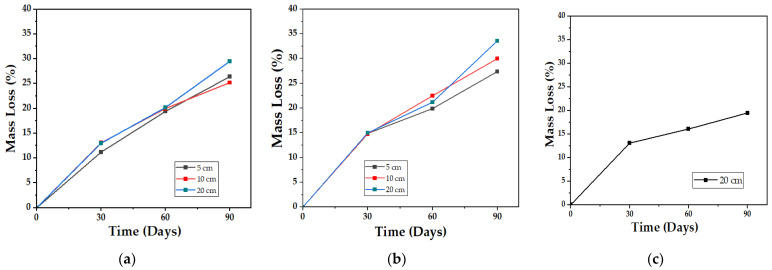
Mass loss profile for the biocomposite (TPS/PCL/F) over a period of 90 days in the degradation media: (**a**) Soil; (**b**) Compost; and (**c**) Water.

The gravimetric analysis shows a gradual loss of mass, with an upward trend as time progresses, with compost being the media with the highest values obtained (34%) after comparing the respective curves at 5, 10, and 20 cm depths, while in the aqueous media, the loss of mass reached does not exceed 20% after 90 days of testing (Figure 15). This was to be expected, considering that PCL takes 2 to 4 years to achieve complete degradation in an aqueous media (hydrolytic degradation) [47] and that the biocomposite is composed of a majority proportion of this polymer (54%). The synergistic effect caused by the presence of bacteria and fungi in the biocomposite is what allows the degradation carried out on land to have higher kinetics.

Mass losses can be attributed to a greater extent to the disappearance of the starch that is part of the biocomposite, since this is a more bio-susceptible material than PCL [48], and although its plasticization with glycerol reduces water absorption and gives it better mechanical properties, with the passage of time, the retrogradation and the decrease in free volume reduce its structural stability and make it fragile [58]. Consequently, two different effects are produced in the biocomposite: On the one hand, when the sample fractures, the surface area increases and more spaces are generated for fungi, bacteria, and even mites to degrade the organic matter, as was recorded in the study of aerobic biodegradation of starch plasticized with glycerol, carried out by Merchán J. et al. [59], where samples of equal mass, but with 4.65 times more surface area, achieved 3 times more degradation than those of smaller area in the same test time. On the other hand, the recrystallization of the material with time (mainly TPS) also makes it less prone to hydrolyze with moisture and hinders assimilation by microorganisms since the retrograded starch is less accessible to amylases, and digestion ceases or decreases [60]; according to [61], the crystallinity of starch samples increases with storage time, so that, as the test days pass, the degradation process slows down.

#### 4.2.5. Statistical Analysis

**Figure 16:**
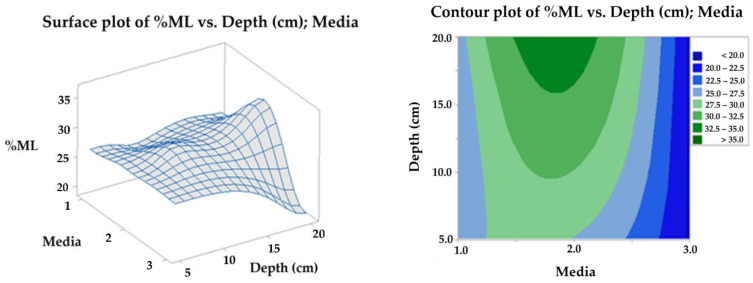
Surface and contour plot for TPS/PCL/F mass loss in degradation media at depth levels.

**Figure 17:**
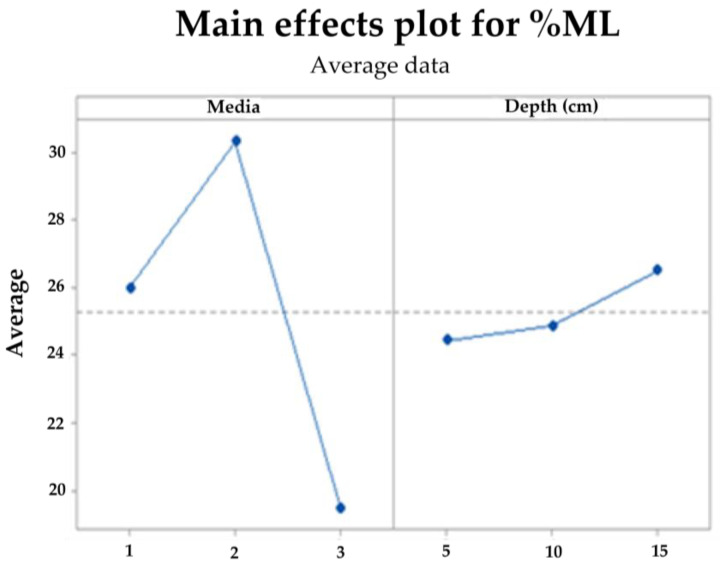
Main effects plot for TPS/PCL/F mass loss in degradation media at depth levels.

With the mass loss data, an analysis of variance (ANOVA) was performed with a confidence level of 95%, comparing the results at 90 days for the three levels, soil, compost, and water (media 1, 2, and 3, respectively). Due to the mixture of effects contributing to degradation, there is a significant difference between the levels evaluated in soil (soil and compost) and those of the aqueous media, whose mass loss does not exceed the mean of 20%, this being the worst scenario for the final disposal of the biomaterial. In contrast, the best result was achieved with the compost at 20 cm depth, followed by the same media at 10 cm, as shown in the contour plots surface (Figure 16) and main effects (Figure 17) for the response variable. The above effect is probably a consequence of the higher microbial activity in media 2, as can be seen in Figure 3, together with its clay characteristics.

## 5. Conclusions

The degradation of the biocomposite based on TPS, PLC, and F of composition 36, 54, and 10%, respectively, was evaluated by subjecting it to different natural environments to simulate the conditions to which the material would be exposed once its useful life cycle is over. The samples evaluated showed signs of degradation from the first 30 days of testing, decreasing their initial mass and losing mechanical properties with respect to the reference values of day 0. At the end of the total time of the experiment, the biocomposite lost, on average, 26, 33, and 20% of its initial mass in the soil, compost, and water media, respectively. Additionally, mechanical properties, such as maximum strength and tensile modulus of elasticity, decreased considerably.

The SEM micrographs obtained on the biocomposite corroborated the surface deterioration of all the samples; the infrared spectra showed that the most bio-susceptible material of the biocomposite was TPS, since the signals corresponding to the main functional groups of starch decreased in intensity to a great extent for all depths. PCL showed greater permanence in the different environments.

The tensile test performed on the biocomposite, as well as the SEM micrographs, corroborated the deterioration of the biocomposite due to changes in its mechanical properties, decreasing the modulus of elasticity and maximum resistance, since the loss of mass and the deterioration on the surface of the biocomposite can make it susceptible to retrogradation, affecting its quality and behavior over time.

Finally, the best degradation conditions for the material were found in the compost media at 20 cm depth, followed by soil, and finally in the aqueous media, which is due to the synergistic effect produced by bacteria, mite fungi, and the humidity conditions to which they are subjected on land (soil and compost), while in water, the mechanisms for decomposing the biomaterial decrease and slow down the process. From the tests, it is also concluded that the degradation process is not linear, since in the mass loss, tensile test, and modulus of elasticity graphs, the slope varies as time goes by, being this variation smaller month by month. Nevertheless, it would be required to extend the time of the experiment to obtain the time required for the total degradation of the evaluated material. The results obtained in this study show a potential approach for the sustainable process to produce biodegradable biocomposite.

## Figures and Tables

**Table 1:** Factors and levels in the experimental design.

Factor	Level
Degradation media	Soil
	Compost
	Water *
Depth	5 cm
	10 cm
	20 cm

Note: * Additional level; only 20 cm was studied in the experimental setup.

**Table 2:** Physicochemical characterization of soil and compost.

Parameter		Sample		Method
		Soil	Compost	Gómez, I.D. [33]
Humidity (%)		24.19	11.42	Gravimetric
pH		5.51 ± 0.07	4.75 ± 0.04	Potentiometric
Electric Conductivity (μS/cm)		95.1 ± 5.3	228.5 ± 3.5	Conductivity meter
Total Nitrogen (%)		1.096 ± 0.001	0.46 ± 0.07	Kjeldahl
Total Organic Matter (%)		5.1 ± 0.5	12.0 ± 0.1	Colorimetric
Salinity (dS/m)		0.095 ± 0.005	0.230 ± 0.004	Conductivity meter
Soil Texture (%)	Sand	38	66	Bouyoucos
	Clay	38.2	16.4	
	Silt	23.8	17.6	

**Table 3:** Physicochemical characterization of the lake water.

Parameter	Valor	Method
		Chacon, M.Y. [34]
pH	6.62 ± 0.05	Potentiometric
Electric Conductivity (μS/cm)	20.8	Conductivity meter
TDS (mg/L)	13.52	Potentiometric
COD (mg/L O_2_)	0.55	Colorimetric
ORP (mV)	598.2	Potentiometric
DO (mg/L)	6.56	Potentiometric
Mesophiles (CFU/mL)	>300	Plate count
Total coliforms	Presence	Presence–Absence
*E. coli*	Presence	Presence–Absence

**Table 4:** Characteristic bands and binding type for PCL, TPS, and the initial sample of TPS/PCL/F biocomposite.

No.	Reference	Wave Number (cm^−1^)
1	Symmetric and asymmetric stretching of methylene groups (CH_2_)	2942
2		2868
3	Carbonyl group stretching (C=O)	1721
4	Hydroxyl bond stretching vibration (-OH)	3291
5	Symmetric and asymmetric stretching of methylene groups (CH_2_)	2930
6		2880
7	Hydroxyl bond bending vibration (-OH)	1645
8	Hydroxyl bond stretching vibration (-OH) (moved)	3335
9	Symmetric and asymmetric stretching of methylene groups (CH_2_)	2941
10		2865
11	Carbonyl group stretching (C=O)	1721
12	Hydroxyl bond bending vibration (-OH)	1645
13	Stretching of starch glycosidic bonding (C-O-C)	1018

## Data Availability

Not applicable.

## Supplementary Materials

The following supporting information can be downloaded at: https://www.mdpi.com/article/10.3390/polym15193952/s1.
