# Supplementary material for: Study of the Degradation of a TPS/PCL/Fique Biocomposite Material in Soil, Compost, and Water
Source: Polymers (Basel). 2023 Sep 30;15(19):3952. doi: 10.3390/polym15193952 (PMC10575001; doi:10.3390/polym15193952)
Supplement: Supplementary file 1 [file polymers-15-03952-s001.zip › polymers-2612381-supplementary.pdf]

Supplementary material

Table S1. Soil pH classification for two determination methods [33].

pH Determined in Water 1:1		pH Determined in KCl 1:1, P:V	
**Value**	**Classification**	**Value**	**Classification**
< 3.5	Ultra acidic	< 4.0	Extremely acidic
3.5 – 4.4	Extremely acidic	4.0 – 4.9	Strongly acidic
4.5 – 5.0	Very strongly acidic*	5.0 – 5.9	Moderately acidic
5.1 – 5.5	Strongly acidic**	6.0 – 6.9	Slightly acidic
5.6 – 6.0	Moderately acidic	7.0	Neutral
6.1 – 6.5	Slightly acidic	7.1 – 8.0	Slightly alkaline
6.6 – 7.3	Neutral	8.1 – 9.0	Moderately alkaline
7.4 – 7.8	Slightly alkaline	9.1 – 10.0	Strongly alkaline
7.9 – 8.4	Moderately alkaline	> 10.1	Extremely alkaline
8.5 – 9.0	Strongly alkaline		
> 9.0	Very strongly alkaline		

Note: Classification in which the *compost and **soil samples are found.

Table S2. Soil type classification according to electrical conductivity [38].

Soil Type	Electrical conductivity (dS/m)
Not saline*	0 – 2
Slightly saline	2 – 4
Moderately saline	4 – 8
Strongly saline	8 – 16
Extreme salinity	> 16

Note: Classification in which *compost and soil samples are found.

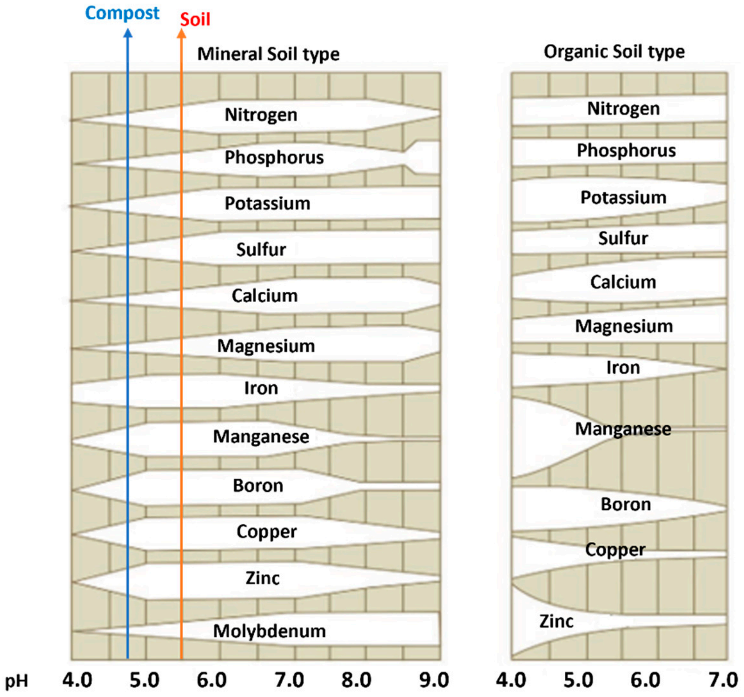

Figure S1. Influence of pH on nutrient solubility in mineral soil and an organic substrate [38].

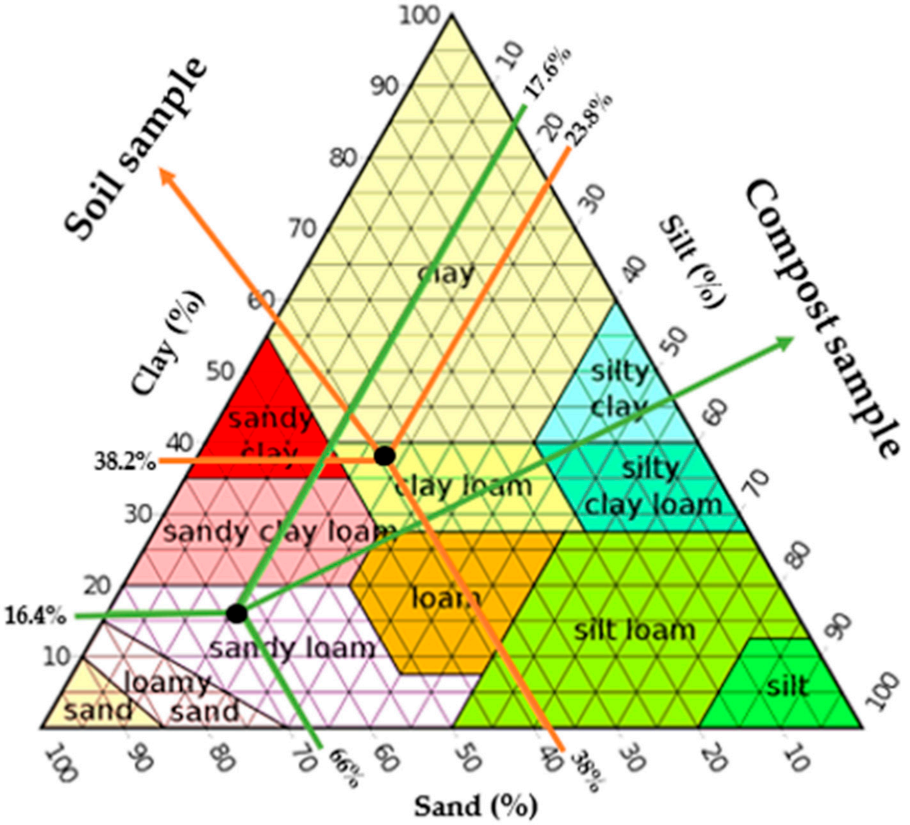

Figure S2. Texture triangle, according to USDA classification.
